# Novel Hydrogels of Chitosan and Poly(vinyl alcohol) Reinforced with Inorganic Particles of Bioactive Glass

**DOI:** 10.3390/polym13050691

**Published:** 2021-02-25

**Authors:** O. Sánchez-Aguinagalde, Ainhoa Lejardi, Emilio Meaurio, Rebeca Hernández, Carmen Mijangos, Jose-Ramon Sarasua

**Affiliations:** 1 Department of Mining-Metallurgy Engineering and Materials Science and POLYMAT, School of Engineering, University of the Basque Country (EHU-UPV), Plaza Ingeniero Torres Quevedo 1, 48013 Bilbao, Spain; oroitz.sanchez@ehu.eus (O.S.-A.); emiliano.meaurio@ehu.eus (E.M.); jr.sarasua@ehu.eus (J.-R.S.); 2 Instituto de Ciencia y Tecnología de Polímeros, CSIC, c/Juan de la Cierva 3, 28006 Madrid, Spain; rhernandez@ictp.csic.es (R.H.); cmijangos@ictp.csic.es (C.M.)

**Keywords:** biodegradable, thermosensitive, rheological properties, bioactivity, drug release

## Abstract

Chitosan (CS) and poly(vinyl alcohol) (PVA) hydrogels, a polymeric system that shows a broad potential in biomedical applications, were developed. Despite the advantages they present, their mechanical properties are insufficient to support the loads that appear on the body. Thus, it was proposed to reinforce these gels with inorganic glass particles (BG) in order to improve mechanical properties and bioactivity and to see how this reinforcement affects levofloxacin drug release kinetics. Scanning electron microscopy (SEM), X-ray diffraction (XRD), swelling tests, rheology and drug release studies characterized the resulting hydrogels. The experimental results verified the bioactivity of these gels, showed an improvement of the mechanical properties and proved that the added bioactive glass does affect the release kinetics.

## 1. Introduction

Hydrogels are polymeric materials with the ability to entrap large amounts of water and maintain a three-dimensional structure formed by interconnected flexible polymer chains [1]. In particular, hydrophilic, water insoluble and elastic hydrogels are suitable for a wide range of applications, including biomedical applications [2,3]. Due to their ability to simulate biological environments, they are receiving a great amount of interest for tissue engineering [4,5]. In addition, these hydrogels are appropriate for controlled drug release systems, being able to administer a drug in a constant way in the organism [6,7]. This is possible thanks to their swelling properties, as their three-dimensional size increases with water absorption, enabling the release of the drug through the polymeric network.

Among the latest research performed on hydrogels, different bioinks based on lignocellulosic biomaterials for 3D bioprinting [8] can be found, with characteristics such as shape integrity [9,10] or control of electrical conductivity [11]. Additionally, through the use of 3D-printing technology, it has been possible to create models of tumors [12] of cardiac microtissues [13] in order to be able to know the effects of drugs on them. In addition to bioprinting, advances can also be seen in the use of hydrogels for drug release, as in the work by Abedi et al., in which a simultaneous release of two drugs is achieved using nanogels [14].

This study is focused on chitosan/poly(vinyl alcohol) hydrogels. Chitosan (CS) is a semicrystalline aminopolysaccharide obtained by deacetylation of chitin. Owing to properties such as nontoxicity, antimicrobial capability [15], biodegradability and excellent biocompatibility, it is being widely used in biomedical and pharmaceutical fields. These applications include tissue engineering [16], drug delivery systems [17], wound healing [18,19], nanoparticle carriers [20] or coatings for implants [21]. In recent research, the most common application of chitosan-based hydrogels has been for drug release, either directly by topical application [22] or in matrices for drug-charged microparticles [23] or for drug-loaded liposomes, avoiding the initial burst and prolonging the duration of the release [24]. Apart from drug release, applications such as scaffolds for cell differentiation can be seen, such as in the work of Amiryaghoubi et al. for human dental pulp stem cells to differentiate into osteoblasts [25], nanocomposite hydrogels for the detection of cancer cells [26] or even as biomechanical energy harvesters to supply power to wearable sensors and electronic devices [27].

Poly(vinyl alcohol) (PVA) is a semicrystalline synthetic polymer of vinyl acetate and vinyl alcohol. It is a suitable material for biomedical applications [28] such as wound dressings [29] or nanoparticle fabrication [30] due to its properties. It is water soluble, nontoxic and biocompatible.

The combination of both polymers results in a hydrogel with a three-dimensional network formed through physical interactions, avoiding the use of crosslinking agents. One of the characteristics to highlight of these hydrogels is their thermosensitivity [31]. This property enables them to change from a sol to gel state when it reaches the transition temperature, which is 37 °C in this case, close to body temperature [32]. Thanks to this characteristic, its use as an injectable liquid which transforms into a semisolid hydrogel is possible [33,34].

Although they have many advantages, the mechanical properties of these hydrogels are insufficient to withstand some loads that can occur in certain areas of the body, such as joints. Therefore, it was proposed to add inorganic particles of bioactive glass (BG) in order to improve these properties [35]. In addition to improving the mechanical properties, bioactive glass is a class A bioactive material; that is, it is osteoconductive and osteoinductive. Moreover, it can not only form bonds with bone tissues, but it can also form chemical and biological bonds with soft tissues [36]. The formation of these bonds is based on the fact that the products of the bioactive glass solution have a direct effect on the deposition of an apatite layer on the surface of the material and on the genetic expression of the surrounding cells [37]. Consequently, there is better integration with the surrounding tissues, prolonging the period that can remain implanted without causing rejection reactions.

**Scheme 1:**
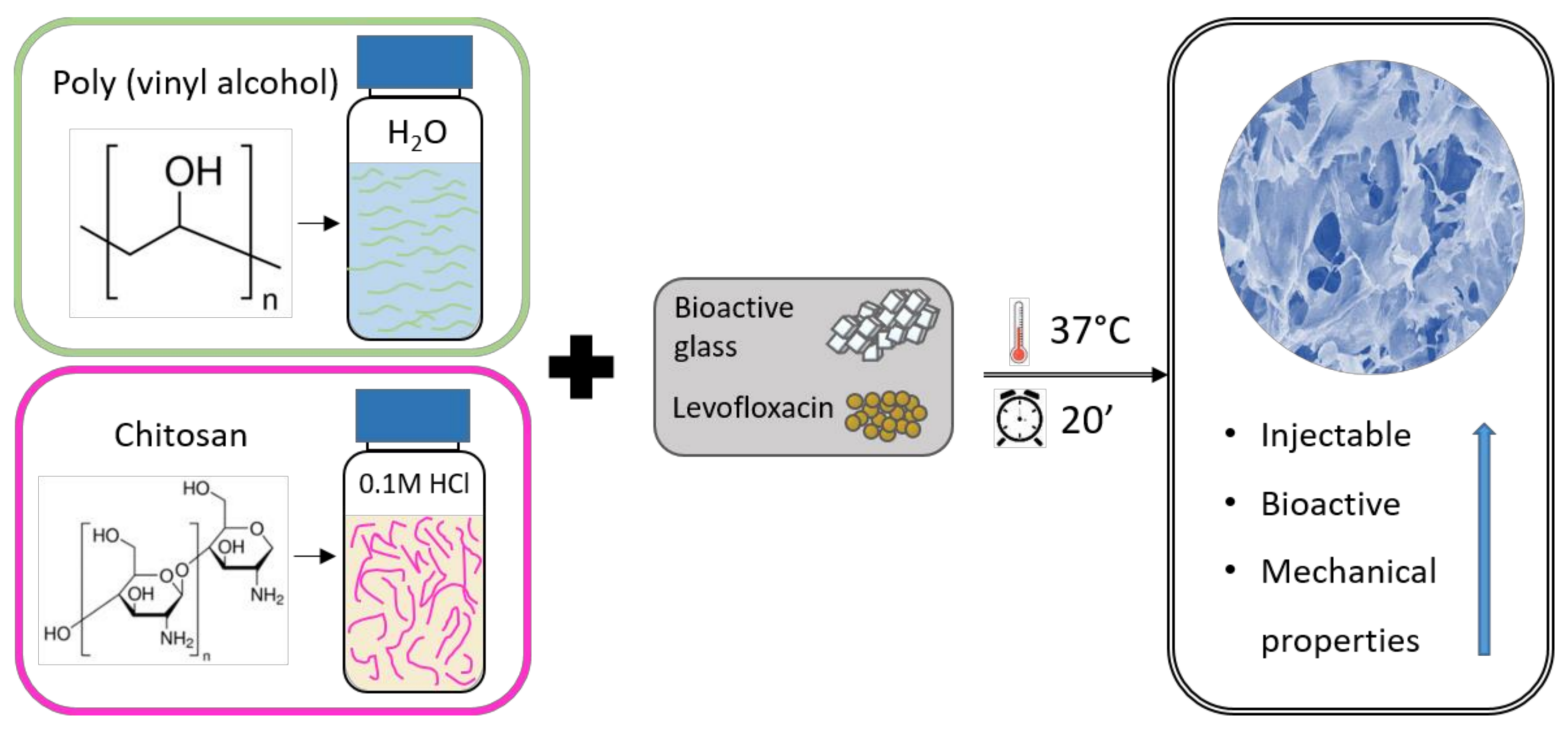
Preparation and aim of the work.

In the present work, chitosan and PVA hydrogels with different amounts of bioactive glass were developed, with the aim of studying their effect on the mechanical properties, bioactivity and drug release of the resulting hydrogels. These hydrogels were prepared by blending PVA with chitosan at two different concentrations followed by addition of the inorganic nanoparticles (as shown in Scheme 1). The swelling and rheological properties were determined as a function of chitosan concentration and bioactive glass content. The bioactivity was determined through the observation of the formation of an apatite layer on the surface of gels by scanning electron microscopy (SEM) and the determination of the crystalline structure as a function of time of immersion of gels in simulated body fluid (SBF) at 37 °C. Finally, the release kinetics of a model drug, levofloxacin, were determined. This fluoroquinolone is an inexpensive, broad-spectrum antibiotic. A good solubility in water makes the dissolution of the drug in PVA aqueous solution fast, and it has high stability. It is easily identifiable by UV spectroscopy, with an absorption peak at 288 nm [38,39]. 

## 2. Materials and Methods

### 2.1. Materials

Poly(vinyl alcohol) (PVA, *M*_w_ = 13000–23000 g/mol, 98% hydrolyzed), chitosan (CS) of low molecular weight (degree of deacetylation = 75%), sodium bicarbonate (NaHCO_3_), phosphate buffered saline (PBS), hydrochloric acid (HCl) and levofloxacin (C_18_H_20_FN_3_O_4_, *M*_w_ = 316.37 g/mol) were supplied by Sigma Aldrich (St. Louis, MO, USA) and used as received.

Bioactive glass was purchased from NovaBone Products, LLC (Alachua, FL, USA) and used as received, and distilled water was provided by Iberia Agua (Zaragoza, Spain). 

### 2.2. Preparation of Chitosan/PVA Hydrogels

Hydrogels were prepared using the method described by Tang et al. [40]. A 2% (*w*/*w*) CS solution was obtained by dissolving chitosan in 0.1 M HCl, and it was stored in the fridge. A 0.5% (*w*/*w*) PVA aqueous solution was obtained by adding PVA to distilled water under magnetic stirring at 80 °C for one hour. To prepare the hydrogel, 0.3 mL of 1 M NaHCO_3_ was added to 3 mL of 0.5% (*w*/*w*) PVA and kept in an ice bath under magnetic stirring for 5 min. This mixture was slowly added to 3 mL of 2% (*w*/*w*) CS solution in an ice bath and kept under magnetic stirring for 2 minutes. Then, bioactive glass was added and maintained under magnetic stirring in ice bath until homogeneous dispersion was achieved. Each solution was distributed in 3 molds of 1.5 mL. The gels were then formed by putting them in the oven at 37 °C. 

Different samples were prepared with varying concentrations of bioactive glass (0% (*v*/*v*), 0.5% (*v*/*v*) and 1.5% (*v*/*v*)) at two CS concentrations (2% and 4% (*w*/*w*)). The designation of the different samples under study is reported in Table 1, as well as the average dry weight of each sample used for the experiments.

### 2.3. Swelling Properties

Samples were lyophilized and immersed in distilled water (50 mL) at room temperature. Average weights of the dry samples of different compositions are indicated in Table 1. At various time intervals, the excess surface water was removed with filter paper, and the swollen samples were weighed. This procedure was repeated until the equilibrium swelling of the samples was reached. With the obtained data, swelling index (SI) (Equation (1)) and equilibrium water content (EWC) (Equation (2)) were determined, using the following equations:

(1) $$Swelling\;ratio\;=\;\frac{Swollen\;weight\;of\;the\;sample}{Dry\;weight\;of\;the\;sample}$$

(2) $$EWC\;\left(\%\right)\;=\;\frac{Weight\;of\;water\;in\;the\;gel}{Total\;weight\;of\;the\;hydrated\;gel}\;\times 100$$

The experiment was repeated with three different samples to ensure reproducibility.

### 2.4. Rheological Measurements

The rheological characterization of the hydrogels was performed in a TA Instruments AR-G2 Rheometer (New Castle, DE, USA) using a steel plate with crosshatched geometry (20 mm diameter). Aqueous solutions were poured in Teflon molds (20 mm diameter) and maintained overnight at 37 °C for sol-to-gel transition. A strain sweep test was carried out to determine the linear viscoelastic range. Frequency sweep experiments were performed over the frequency range of 0.01–100 Hz. All experiments were carried out at 37 °C and at a constant strain of 1% located within the linear viscoelastic region. The elastic modulus (G′) and viscous modulus (G′′) were calculated using the Rheology Advantage Data Analysis Software. The experiment was performed in triplicate for each sample.

### 2.5. Bioactivity Studies

For the study of the in vitro bioactivity of the prepared samples, PVA/CS 2, PVA/CS/BG 2/0.5 and PVA/CS/BG 2/1.5 were immersed in SBF at 37 °C and extracted at different periods (0, 7, 14 and 28 days). Then, they were washed and frozen in order to lyophilize them and obtain dehydrated samples. In order to maintain the concentration of cations throughout the whole experiment, the SBF was renewed every 7 days [37]. 

In addition, 0.5 L of simulated body fluid (SBF) was prepared by dissolving 4.0145 g sodium chloride (NaCl) purchased from Panreac Química S.A.U. (Barcelona, Spain), 0.1775 g sodium bicarbonate (NaHCO_3_), 0.1125 g potassium chloride (KCl), 0.1155 g potassium hydrogen phosphate trihydrate (K_2_HPO_4_·3H_2_O), 0.1555 g magnesium chloride hexahydrate (MgCl_2_·6H_2_O), 0.146g calcium chloride (CaCl_2_) and 0.036 g sodium sulfate (Na_2_SO_4_); all of them were acquired from Sigma Aldrich and pH was adjusted to 7.4 using hydrochloric acid (HCl) and 3.059 g tris (hydroxymethyl aminomethane) [(CH_2_OH)_3_CNH_2_] obtained also from Panreac Química S.A.U.

The formation of an apatite layer on the surface of the hydrogel was observed through scanning electron microscopy (SEM) performed on dehydrated samples using a Hitachi S-4800 (Tokio, Japan), after coating them with a 15 nm gold layer in an Emitech k55x Sputter Coater (Montigny, France) at 25 mA.

An X-ray diffraction (XRD) analysis was performed to confirm the formation and crystallization of the apatite layer. A Phillips X’pert Pro diffractometer (Amsterdam, The Netherlands) was used operating at 40 kV and at 40 mA, in a theta-theta configuration, with a Cu anode and a PSD detector. The scanning scope of 2θ was 10–50°, with a step size of 0.026° and step time of 148.92 s at 25 °C.

Experiments were repeated with three different samples to ensure reproducibility. 

### 2.6. Drug Release

For the study of the drug release from PVA/CS hydrogels, levofloxacin was employed as a model drug. Levofloxacin (5 mg/mL) was dissolved in PVA/CS solutions by magnetic stirring, and then the chitosan/PVA gels were prepared following the same procedure previously described. Each of the samples carried a total of 7.5 mg of drug. Gels were immersed into 100 mL of 0.1 M phosphate buffered saline (PBS), pH 7.4, at 37 °C, and aliquots of 0.2 mL were taken out at regular time intervals. The release of levofloxacin was determined by UV spectrophotometry at 288 nm (Perkin Elmer Lambda 265) (Waltham, MA, USA). 

The kinetics of the release from the different samples were determined by finding the best fit of the curves to different kinetic models. Four different mathematical models were considered:

(3) $$\mathrm{Zero}\;\mathrm{order}:\;C_{t}/C_{\infty }=k_{0}t$$

(4) $$\mathrm{First}\;\mathrm{order}:\;\ln\left(1-C_{t}/C_{\infty }\right)=-k_{1}t$$

(5) Higuchi: Ct/C∞=kht12

(6) $$\mathrm{Korsmeyer}–\mathrm{Peppas}:\;C_{t}/C_{\infty }=kt^{n}$$

*C_t_* is the cumulative amount of drug released at time *t, C*_∞_ is the starting amount of drug, *n* is the release exponent and *k*_0_, *k*_1_, *k_h_* and *k* are the kinetic constants. Zero-order kinetics represent a release process that is controlled by relaxation of polymeric chains, with a constant release rate of drug, independent of its concentration (Equation (3)). On the other hand, a drug release rate that depends on its concentration is represented by a first-order kinetics model (Equation (4)) [41]. In the case of Higuchi (Equation (5)), it describes drug release as a diffusion process, square-root time dependent, based on Fick’s law. The last model, the Korsmeyer–Peppas model (Equation (6)), is useful when the release mechanism is not well known or when more than one type of release phenomena could be involved. Depending on the values obtained for the release exponent, *n*, it is possible to define whether if the release happens by Fickian diffusion, anomalous transport, Case-II transport or Super Case-II transport [42].

### 2.7. Statistical Analysis

Data were subjected to one-way analysis of variant (ANOVA) with the level of significance set at *p* < 0.05.

## 3. Results and Discussion

### 3.1. Gel Formation

**Figure 1:**
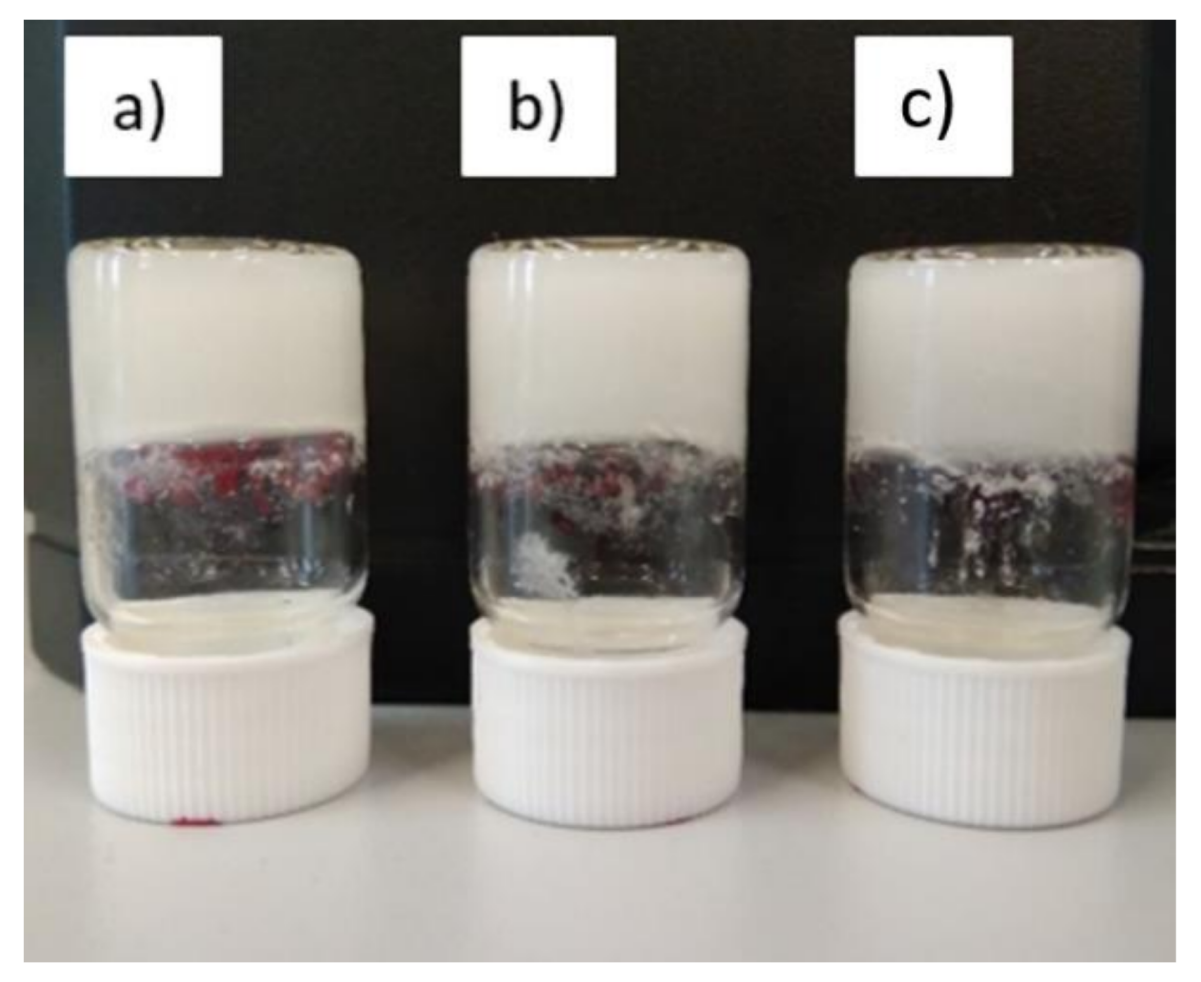
Representative results obtained from inverted vial tests. (**a**) PVA/CS 2, (**b**) PVA/CS/BG 2/0.5 and (**c**) PVA/CS/BG 2/1.5 hydrogels.

To determine gel formation after the addition of bioactive glass, an inverted vial test was employed and the representative images corresponding to samples prepared at 2% *w*/*v* CS concentration are shown in Figure 1. The formation of gel was verified for the samples as the nonflowing gel maintained its position after vial inversion. Similar results were obtained for samples prepared at 4% *w*/*v* CS concentration (results not shown). 

### 3.2. Effect of the Chitosan Concentration and Bioactive Glass Content on the Swelling and Rheological Properties

**Figure 2:**
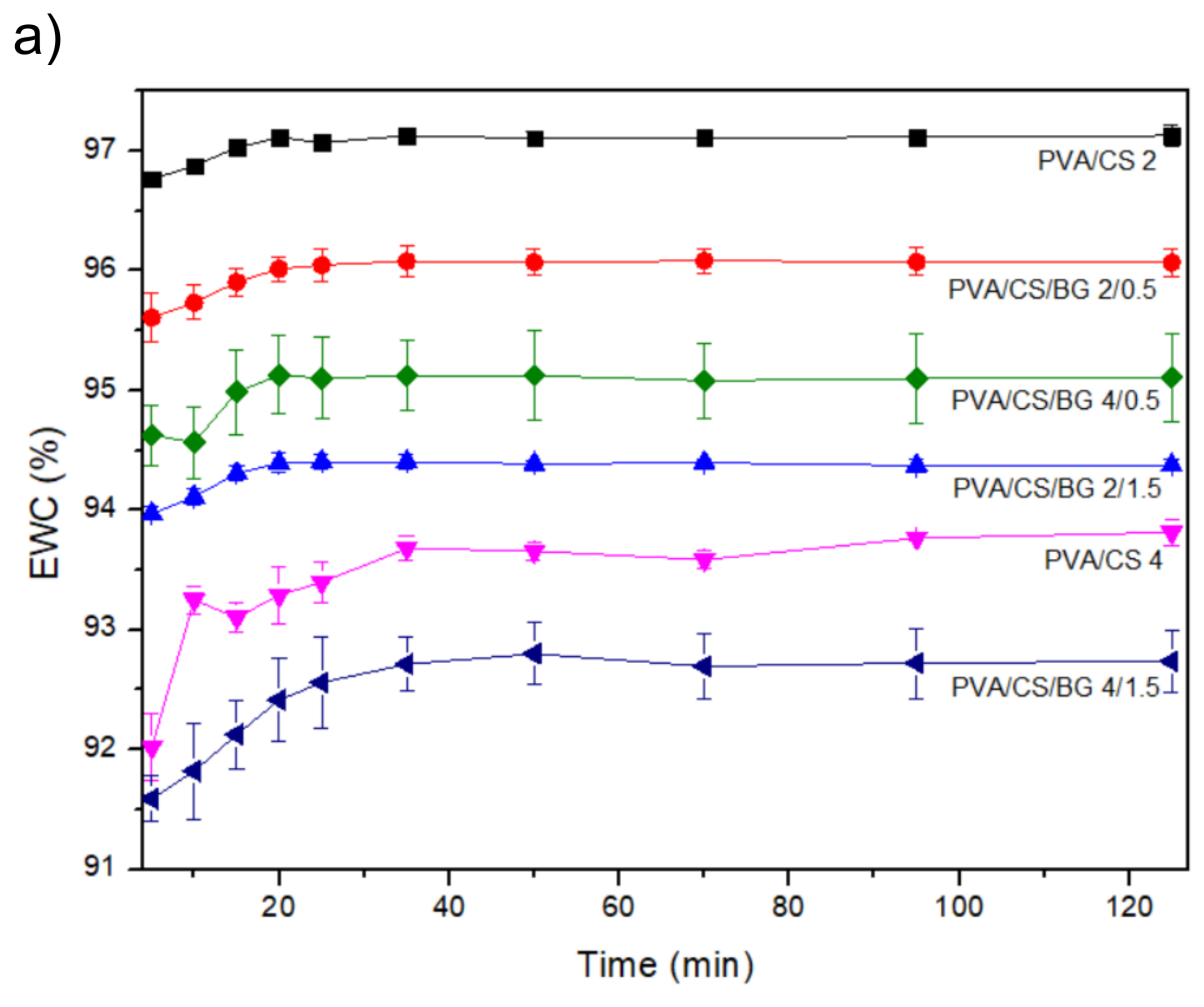

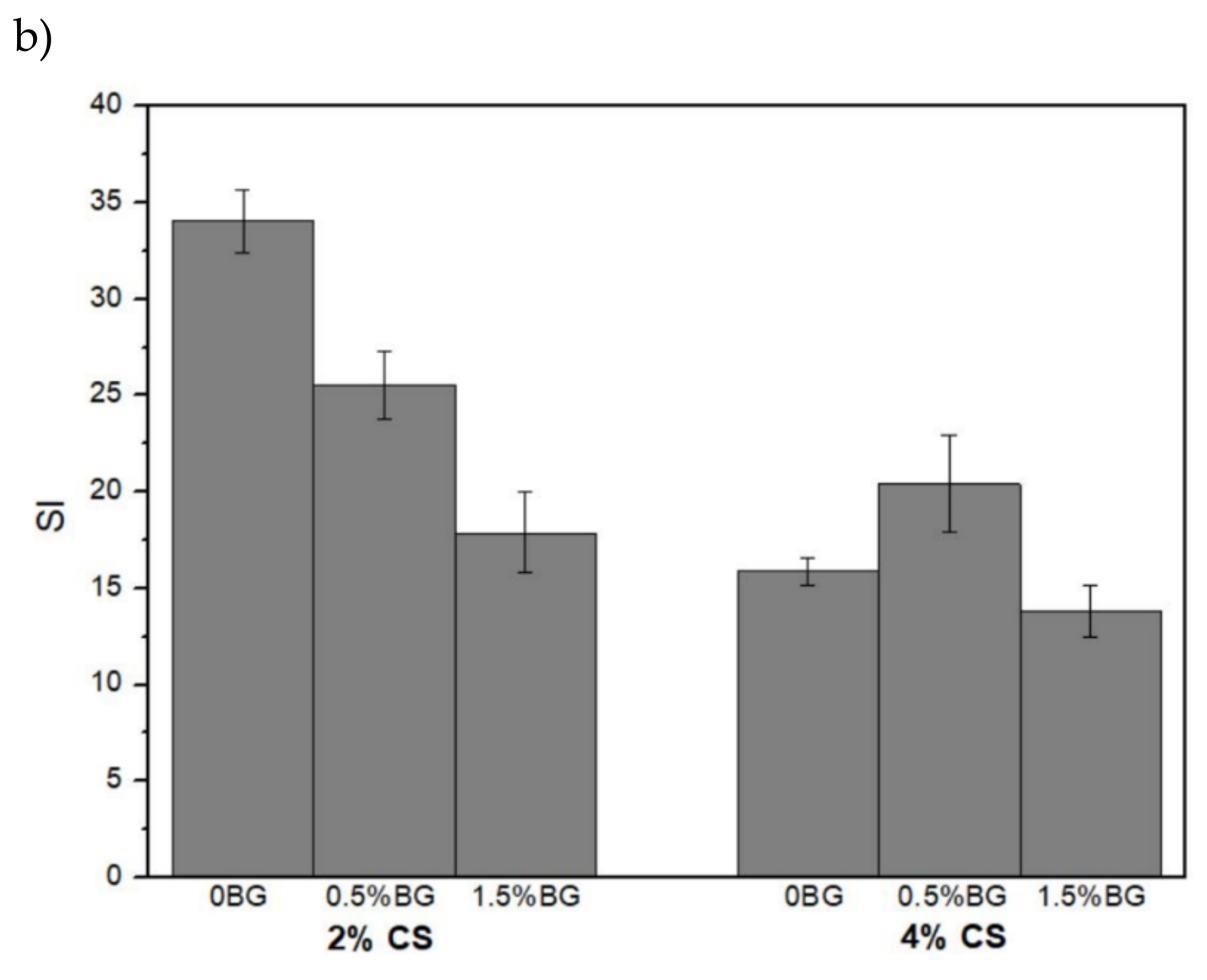
(**a**) Equilibrium water content (EWC) and (**b**) swelling index (SI) of the hydrogels immersed in 50 mL distilled water at room temperature for 2 h.

Figure 2a shows the results corresponding to swelling experiments carried out on the samples under study. As it can be observed, all the samples showed fast swelling, reaching equilibrium after 20 min of immersion in distilled water at room temperature, with equilibrium water contents (EWC) that exceeded 90%. The increase in chitosan concentration caused a decrease of the swelling index (SI) (Figure 2b), which is in agreement with the work of Tang et al. [40], as long as the PVA proportion is kept low. Regarding the effect of the bioactive glass concentration on the swelling properties of the samples under study, except for the case of PVA/CS/BG 4/0.5, a decrease in the swelling ability with the increase in bioactive glass concentration was observed (Figure 2a). This same effect was described in literature and could be the result of retractile forces of the hydrogel caused by the ion release of bioactive glass particles [43] or the lack of empty gaps after the addition of bioactive glass. 

**Figure 3:**
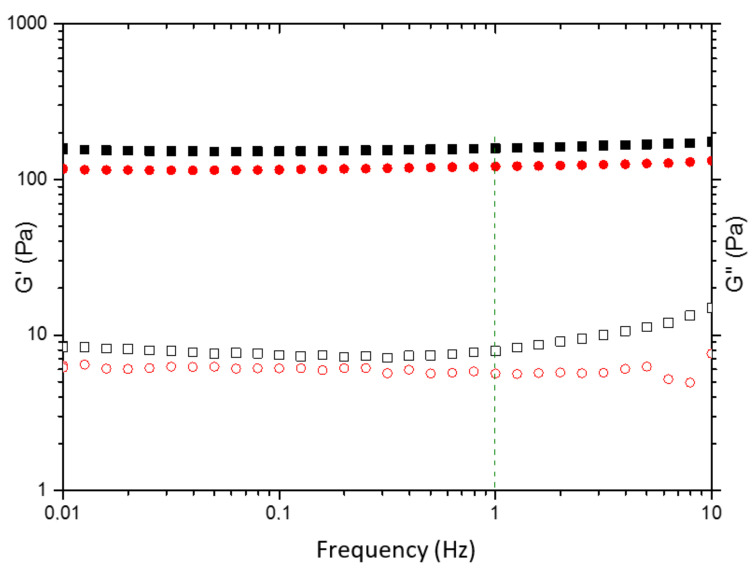
Elastic modulus (**closed symbols**) and loss modulus (**open symbols**) as a function of frequency of PVA/CS 2 (●) and PVA/CS/BG 2/1.5 (■).

The effect of the incorporation of bioactive glass on PVA/CS on the rheological properties of PVA/CS hydrogels was determined by means of oscillatory shear measurements. Figure 3 shows representative results corresponding to frequency experiments carried out on samples PVA/CS 2 and PVA/CS/BG 2/1.5. As it can be observed, both samples present gel-like behavior, which is characterized by the following: (i) values of the elastic modulus are higher than the corresponding viscous modulus (G′ > G″) in the frequency range and (ii) G’ and G″ are independent of frequency in all the ranges under study.

**Figure 4:**
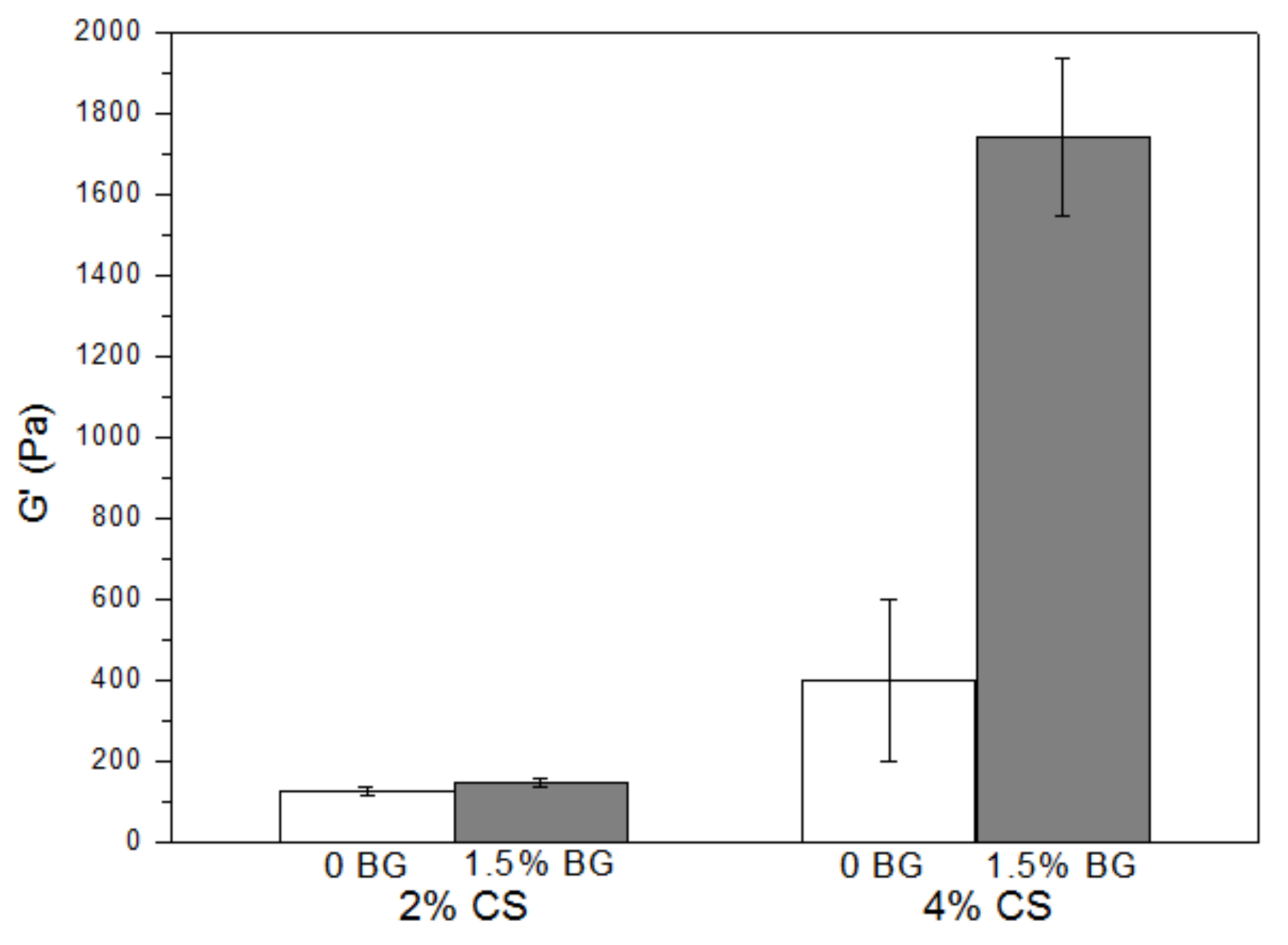
Elastic modulus at frequency = 1 Hz for PVA/CS 2, PVA/CS/BG 2/1.5, PVA/CS 4 and PVA/CS/BG 4/1.5.

The values of the elastic modulus measured at frequency = 1 Hz are depicted in Figure 4 for samples PVA/CS and samples PVA/CS/BG. As observed, the increase in chitosan concentration results in an increase in the elastic moduli obtained for gels without bioactive glass, which can be related to the decrease in the swelling index (SI) [44] found for PVA/CS 4 with respect to sample PVA/CS 2 (Figure 2b). With the incorporation of 1.5% (*w*/*v*) bioactive glass, the elastic modulus measured for PVA/CS and PVA/CS/BG 1.5 hydrogels remains within the experimental error being 129 ± 9 and 149 ± 11 Pa, respectively. Taken into account that the SI determined for PVA/CS/BG 1.5 greatly decreases with respect to PVA/CS, the results might indicate that the incorporation of 1.5% bioactive glass prevents the formation of crosslinking interactions between PVA and CS, leading to the loss of elastic properties of the resulting composite gel [45]. In contrast, the sample PVA/CS/BG 4/1.5 shows an increase in elastic modulus with respect to PVA/CS 4. In this case, and taking into account that the swelling index does not change with the incorporation of bioactive glass, the results obtained could be attributed to the reinforcement effect of micrometric bioactive glass particles onto the polymer matrix as previously reported for other composite gels [46,47]. 

### 3.3. Determination of the Bioactivity

When introducing the hydrogels with bioactive glass in SBF, a layer of apatite must form on the surface, creating both chemical and biological bonds with bone or soft tissue. Observing this layer, it is possible to determine if the samples under study are bioactive.

**Figure 5:**
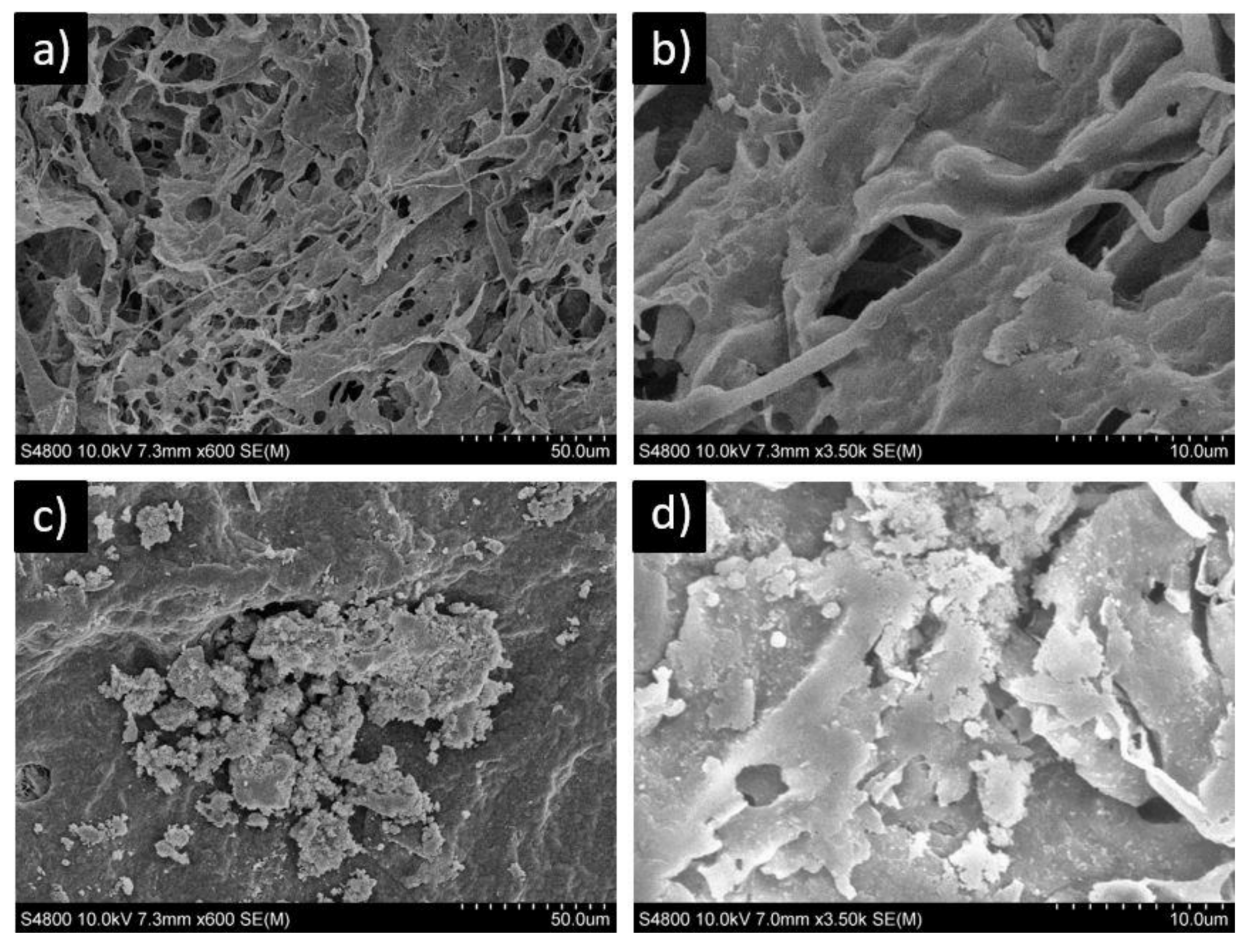
Scanning electron micrographs of PVA/CS 2 at day 0. (**a**) Magnification ×600, (**b**) magnification ×3500) and PVA/CS/BG 2/1.5 after 28 days submerged in SBF, (**c**) magnification ×600 and (**d**) magnification x3500).

The morphology of PVA/CS 2/1.5 hydrogels after being immersed in SBF for 28 days can be observed in Figure 5c,d at two different magnifications (×600 and ×3500). The results obtained are compared to the morphology obtained for PVA/CS (Figure 5a,b). The hydrogel with no BG particles shows a porous morphology typical of a hydrogel. In contrast, the surface corresponding to PVA/CS/BG 2/1.5 hydrogels shows no porosity and seems to be covered by an external layer that presents a cauliflower shape. It is thought that this layer could be the result of the formation of hydroxyapatite from the added bioactive glass, as it has been reported in other works [43,48,49].

**Figure 6:**
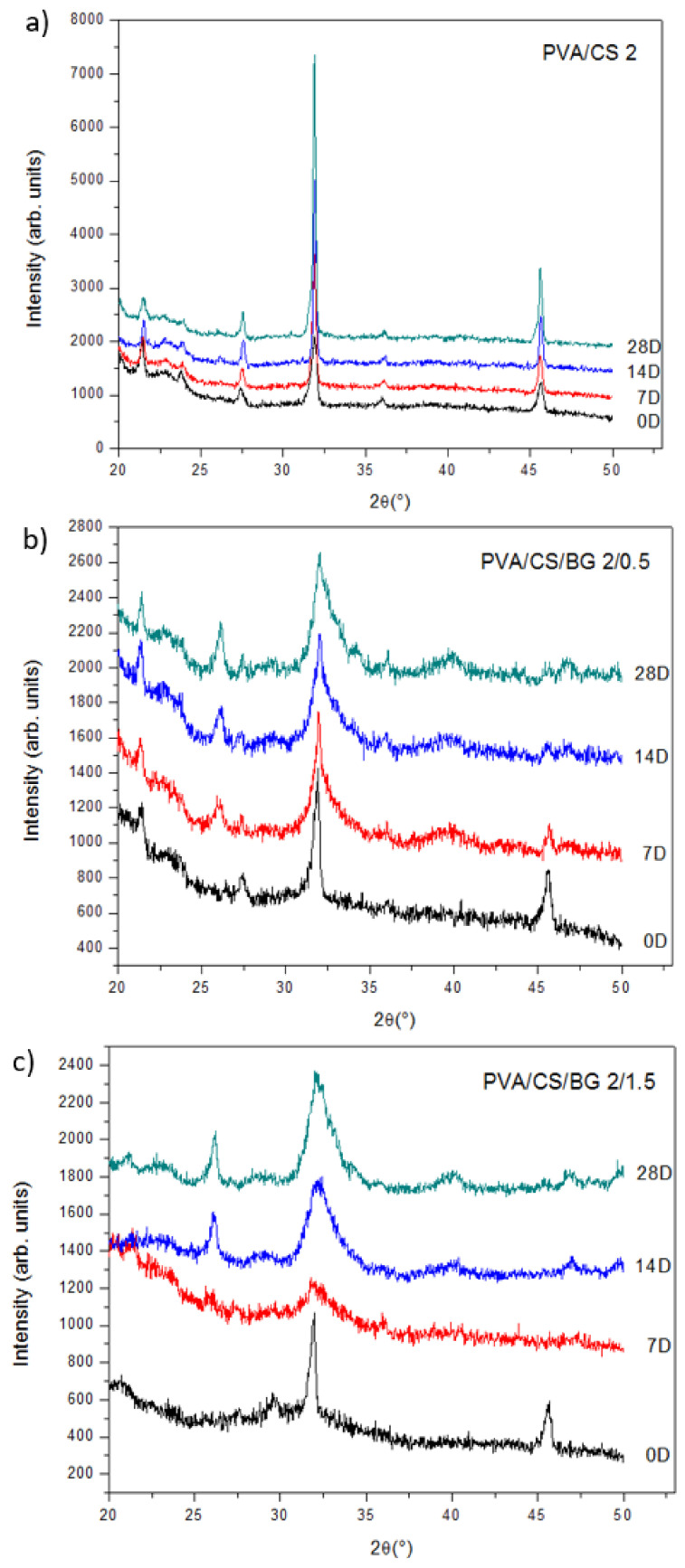
XRD patterns of hydrogels after immersion in SBF for different times. (**a**) PVA/CS 2, (**b**) PVA/CS/BG 2/0.5 and (**c**) PVA/CS/BG 2/1.5.

By means of X-ray crystallography (XRC), the formation of crystalline structures on hydrogels immersed in SBF for different periods of time up to one month was determined. In order to deduce whether there was hydroxyapatite formation or not, special attention was paid to the characteristic peaks of HA found at 2Θ of ~26° and ~32°. In hydrogels without any added bioactive glass (Figure 6a), it was possible to see that there are calcium impurities (~27° and ~32°), and no hydroxyapatite was found. Impurities could be due to insufficient washing of the gels after being immersed in SBF. With the incorporation of 0.5% (w/v) bioactive glass, hydroxyapatite appears almost from the beginning (Figure 6b), increasing over the course of days. This could be ascertained because of the appearance of the diffraction peaks located at ~26° and ~32°. Although the beginning of the peak was more characteristic of calcium, it widened to become more similar to that of hydroxyapatite. Finally, hydrogels with 1.5% (*w*/*v*) concentration of bioactive glass exhibited similar results to those corresponding to hydrogels with 0.5% (w/v) BG (Figure 6c), showing a progressive increase in the peak located at ~26° and the broadening of the peak located at 32°. The diffraction peaks observed in the experiments corresponding to hydrogels with added bioactive glass are wide, which suggests that the size of the crystalline structures is as small as those that can be found in bone [48]. 

XRD experiments allowed confirming that the formation of hydroxyapatite is progressive over time. The reason why there was not much difference between the results found for samples with two different concentrations of bioactive glass (0.5% and 1.5% (*w*/*v*)) could be attributed to the formation of agglomerates. It is extremely difficult to achieve a homogenous dispersion with such a considerable amount of bioactive glass, losing surface due to the agglomerates. The results obtained are in agreement to those reported in literature for hydrogels [43,48,49] and other types of scaffolds with added bioactive glass [37]. 

### 3.4. Study of the Kinetics of Release of a Model Drug

**Figure 7:**
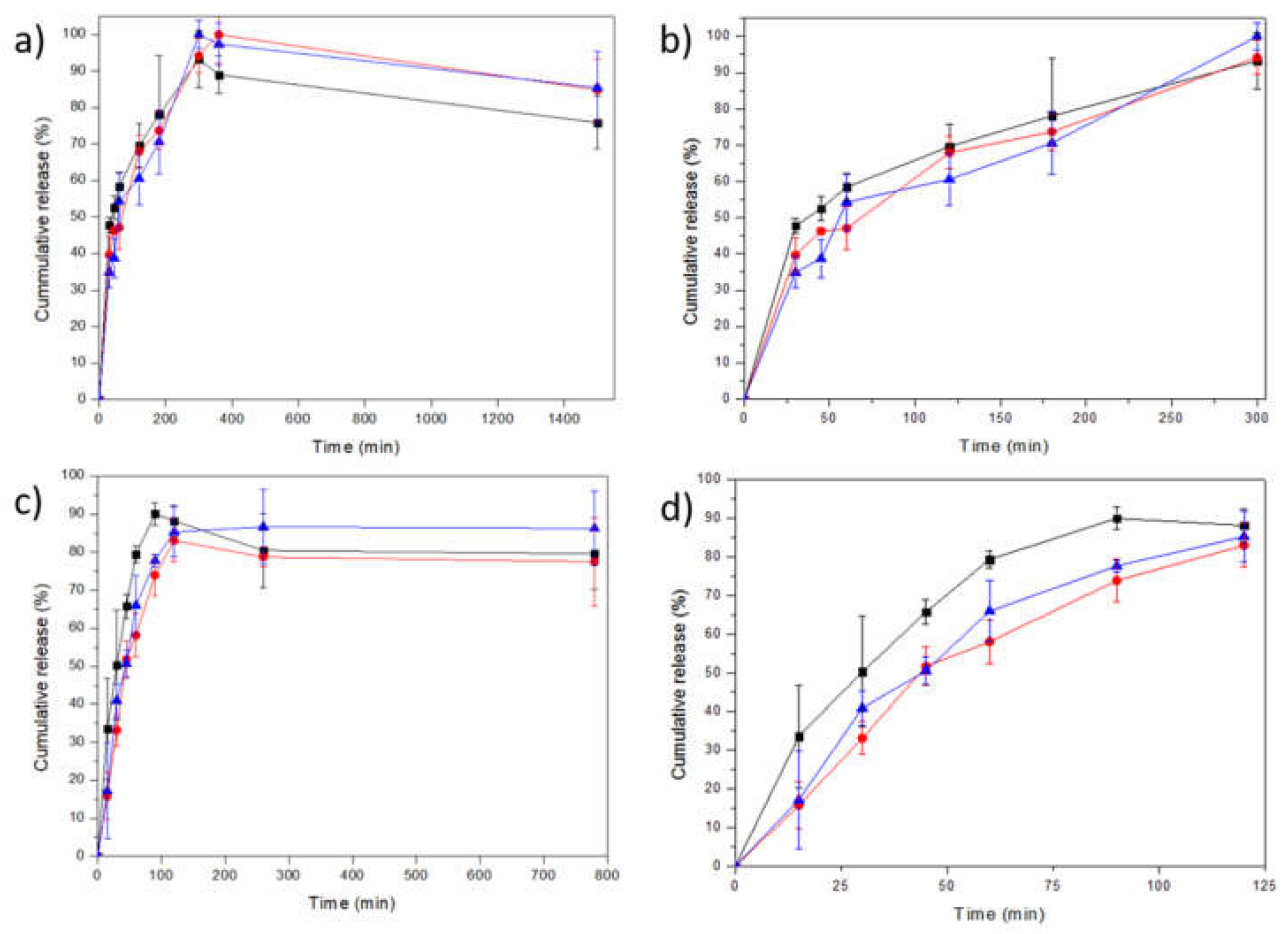
Drug release profile of (**a**) and (**b**) PVA/CS 2 (■), PVA/CS/BG 2/0.5 (●) and PVA/CS/BG 2/1.5 (▲) and (**c**) and (**d**) PVA/CS 4 (■), PVA/CS/BG 4/0.5 (●) and PVA/CS/BG 4/1.5 (▲) Samples immersed in 100 mL of PBS (pH 7.4) at 37 °C for 5 h.

Drug release was tested for all the samples under study, employing levofloxacin as a model drug. For hydrogels with 2% (*w*/*v*) chitosan concentration, the release was almost complete after 5 h regardless of the concentration of bioactive glass (Figure 7a). In fact, no differences were observed between the sample with no added bioactive glass and samples with 0% and 1.5% (*w*/*v*) bioactive glass, which indicates that the release kinetics of levofloxacin are not affected by the presence of BG particles. This may lead us to think that the size of the mesh is large enough in relation to the size of the drug, since despite the addition of the bioactive glass, no great differences are seen in the release, so the drug can continue to travel through the hydrogel easily. On the other hand, hydrogels with 4% (*w*/*v*) chitosan concentration showed a much faster release rate reaching an almost complete release after 2 h (Figure 7b). It is important to note that the amount of drug release was lower with respect to that found for samples with 2% (*w*/*v*) chitosan concentration. In addition, hydrogels with added bioactive glass showed a lower amount of drug release with respect to the hydrogel with no added bioactive glass. These results do not fit with those expected, since a higher polymer concentration leads to a smaller mesh. Therefore, the drug should have more difficulty traveling than hydrogels with 2% (*w*/*w*) CS. However, a larger difference is observed between hydrogels with or without BG; therefore, in this case, it can be deduced that the added bioactive glass is blocking the passage of the drug. 

In order to shed further light on the results obtained, the kinetics of release were fitted to different kinetic equations used to study the mechanism of drug release. Table 2 shows the regression coefficients. PVA/CS 2 and PVA/CS 4, the two systems without added bioactive glass, fit with the Korsmeyer–Peppas model, with correlation coefficients of 0.968 and 0.998, respectively. However, their release exponents show different release mechanisms. The release exponent for PVA/CS 2 is 0.29, too low to fit the mechanisms that were previously mentioned. This could happen due to the pore-size distribution of the matrix, leading to a deviation of the diffusion laws [50]. Analyzing the correlation coefficients of the rest of the models, the release mechanism of this system could be closer to first-order kinetics than to Fick’s law. This is an expected result, since when the drug is transported freely through the mesh, the release depends on the concentration [51]. On the other hand, the release exponent for PVA/CS 4 is 0.61, which indicates abnormal transport, but one that is predominantly controlled by Fick’s diffusion. As mentioned above, although it cannot be seen in the results, in this case the mesh is smaller, so the release could depend on the concentration gradient. PVA/CS/BG 2/0.5 and PVA/CS/BG 2/1.5 systems fit Higuchi model; thus, the release is based in Fick’s law, so the rate of diffusion is directly proportional to a concentration gradient across the membrane [52]. However, PVA/CS/BG4/0.5 and PVA/CS/BG 4/1.5 follow first-order kinetics. In this case, the release depends on drug concentration. This could be due to the difficulty of the drug to go through the matrix when the pore size is smaller, in addition to the obstruction caused by bioactive glass particles. When the drug concentration is higher, there is a larger amount closer to the surface, which makes the release faster.

## 4. Conclusions

In the present work, CS/PVA hydrogels were reinforced with inorganic bioactive glass particles in order to improve their mechanical properties and bioactivity. The effect of the chitosan and bioactive glass concentration on the swelling and mechanical properties was studied. After the satisfactory formation of the hydrogels, it was found that the increase in chitosan concentration results in a lower swelling index for all the samples regardless of the bioactive glass concentration. For samples with 2% (*w*/*v*) chitosan concentration, the addition of BG particles decreases the swelling index without increasing the elastic moduli. This is not the case for the samples prepared with 4% (*w*/*v*) chitosan concentration for which the swelling index is not affected by the addition of BG particles; however, the elastic modulus largely increases with the addition of 1.5% (*w*/*v*) BG particles. The results point, on the one hand, to the decrease in effective crosslinking points in between polymer chains as a result of the BG incorporation, and on the other hand, to a reinforcement effect of the BG particles as previously seen for other composite gels. Bioactivity of the hydrogels containing BG particles was confirmed after immersion of the hydrogels in SBF for one month at physiological temperature, being able to appreciate the formation of a layer of apatite on the surface of hydrogels through SEM carried out on lyophilized samples. XRD further confirmed the formation of crystalline hydroxyapatite. The potential application of CS/PVA hydrogels with inorganic BG particles as drug delivery matrices was also proven by employing levofloxacin as a model drug. The presence of bioactive glass particles does not influence the kinetics of the release in the case of hydrogels with 2% (*w*/*v*) concentration of chitosan; however, it slows the release when the concentration of chitosan is increased to 4% (*w*/*v*) in agreement with the lower swelling index found for these samples. 

## Figures and Tables

**Table 1:** Designation of all samples under study.

Sample Name of the Gel	Ratio between PVA/CS in the Gel	Concentration of PVA (% *w*/*w*) in the Gel	Concentration of CS (% *w*/*w*) in the Gel	Concentration of Bioactive Glass (% *v*/*v*) in the Gel	Dry Weight of Each Sample (mg)
**PVA/CS 2**	50/50	0.5	2	0	14.4 ± 0.3
**PVA/CS/BG 2/0.5**	50/50	0.5	2	0.5	21.7 ± 0.6
**PVA/CS/BG 2/1.5**	50/50	0.5	2	1.5	32.2 ± 0.4
**PVA/CS 4**	50/50	0.5	4	0	23.3 ± 0.7
**PVA/CS/BG 4/0.5**	50/50	0.5	4	0.5	28.7 ± 2
**PVA/CS/BG 4/1.5**	50/50	0.5	4	1.5	44 ± 1.5

**Table 2:** Fitting of the release data to the mathematical models for drug release kinetics. R2 is the correlation coefficient, and n is the release exponent.

	Zero Order	First Order	Higuchi	Korsmeyer–Peppas	
	R^2^	R^2^	R^2^	R^2^	n
**PVA/CS 2**	0.65	0.955	0.92	***0.968***	***0.29***
**PVA/CS/BG 2/0.5**	0.773	0.958	***0.974***	0.933	0.38
**PVA/CS/BG 2/1.5**	0.832	0.872	***0.972***	0.804	0.42
**PVA/CS 4**	0.765	0.901	0.955	***0.998***	***0.61***
**PVA/CS/BG 4/0.5**	0.916	***0.996***	0.961	0.978	0.97
**PVA/CS/BG 4/1.5**	0.888	***0.993***	0.963	0.961	0.96

## Data Availability

The data presented in this study are available in [Novel Hydrogels of Chitosan and Poly(vinyl alcohol) Reinforced with Inorganic Particles of Bioactive Glass.
